# 2-[3-(1*H*-Benzimidazol-2-yl)prop­yl]-1*H*-benzimidazol-3-ium 3,4,5-tri­hydroxy­benzoate–1,3-bis­(1*H*-benzimidazol-2-yl)propane–ethyl acetate (2/1/2.94): co-crystallization between a salt, a neutral mol­ecule and a solvent

**DOI:** 10.1107/S2056989023004279

**Published:** 2023-05-23

**Authors:** José Carlos Palacios Rodríguez, Angel Mendoza, Martha Sosa Rivadeneyra, Sylvain Bernès

**Affiliations:** a Facultad de Ciencias Químicas, Benemérita Universidad Autónoma de Puebla, 72570 Puebla, Pue., Mexico; b Instituto de Ciencias, Benemérita Universidad Autónoma de Puebla, 72570 Puebla, Pue., Mexico; c Instituto de Física, Benemérita Universidad Autónoma de Puebla, 72570 Puebla, Pue., Mexico; Vienna University of Technology, Austria

**Keywords:** crystal structure, co-crystal, hydrogen bonds, supra­molecular structure, solvent mask

## Abstract

The chemical formula of the title compound, 2C_17_H_17_N_4_^+^·2C_7_H_5_O_5_^−^·C_17_H_16_N_4_·2.94C_4_H_8_O_2_, was established by X-ray diffraction of a single-crystal obtained by reacting 1,3-bis­(benzimidazol-2-yl)propane (*L*) and gallic acid (HGal) in ethyl acetate. The mol­ecular structure can be described as a salt (H*L*)^+^(Gal)^−^ co-crystallized with a mol­ecule *L*, with a stoichiometric relation of 2:1. Moreover, large voids in the crystal are filled with ethyl acetate, the amount of which was estimated by using a solvent mask during structure refinement, affording the chemical formula (H*L* ^+^·Gal^−^)_2_·*L*·(C_4_H_8_O_2_)_2.94_. The arrangement of components in the crystal is driven by O—H⋯O, N—H⋯O and O—H⋯N hydrogen bonds rather than by π–π or C—H⋯π inter­actions. In the crystal, mol­ecules and ions shape the boundary of cylindrical tunnels parallel to [100] *via R* (rings) and *D* (discrete) supra­molecular motifs. These voids, which account for about 28% of the unit-cell volume, contain disordered solvent mol­ecules.

## Abstract 2

The title compound can be described as a salt (H*L*)^+^(Gal)^−^ (*L* = 1,3-bis­(benzimidazol-2-yl)propane (*L*); HGal = gallic acid) co-crystallized with a neutral mol­ecule *L.* The crystal also comprises disordered solvent ethyl acetate mol­ecules.

## 1. Chemical context

Bis-imidazole and bis-benzimidazole ligands are frequently used in coordination chemistry because of their chelating properties. Moreover, the size and the nature of the bridge connecting the imidazole moieties can modify the spectroscopic and physicochemical properties of the resulting complexes (Pandiyan *et al.*, 1997 ▸). Such behaviour is useful in bioinorganic chemistry, in particular for the design of models of active centres in metalloproteins. In the specific case of 1,3-bis­(benzimidazol-2-yl)propane (C_17_H_16_N_4_, abbreviated *L* hereafter), coordination complexes with late transition metals have been reported (Co^II^, Ni^II^, Cu^II^, Zn^II^, Ag^I^ and Cd^II^; see for example: van Albada *et al.*, 1999 ▸).

Another salient aspect for these mol­ecules is that they include both acidic protons and protonable sites, allowing the formation of cations or anions, for example by modifying the pH value of the medium. However, the symmetric character of *L* leads to a reasonable assumption that both benzimidazole moieties should behave similarly, so that a dicationic species H_2_ *L* ^2+^ is more readily available compared to the dissymmetric cation H*L* ^+^. We report herein the crystal structure of a compound overriding this rule of thumb, since it contains both neutral *L* and cationic H*L* ^+^ species, together with gallate anions Gal^−^ (3,4,5-tri­hydroxy­benzoate, C_7_H_5_O_5_^−^, derived from gallic acid, HGal) for charge balancing. Moreover, disordered solvent mol­ecules (ethyl acetate, C_4_H_8_O_2_) are present in the crystal, which can then be seen as an uncommon case of a solvated co-crystal between a salt and a mol­ecule. 
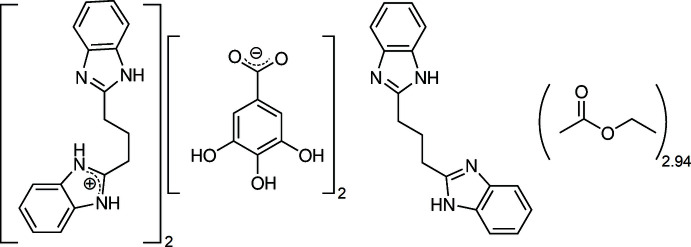


## 2. Structural commentary

**Figure 1:**
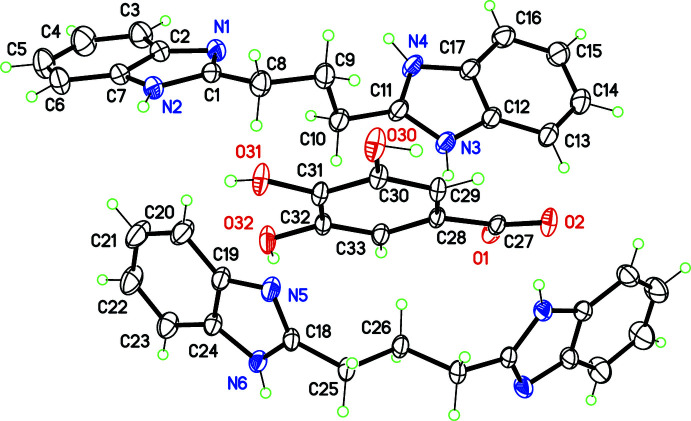
The structures of the mol­ecular entities in the title compound, with displacement ellipsoids for non-H atoms at the 30% probability level. Non-labelled atoms in the neutral moiety (bottom mol­ecule) are generated by symmetry 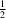 − *x*, *y*, 1 − *z* (twofold rotation).

**Figure 2:**
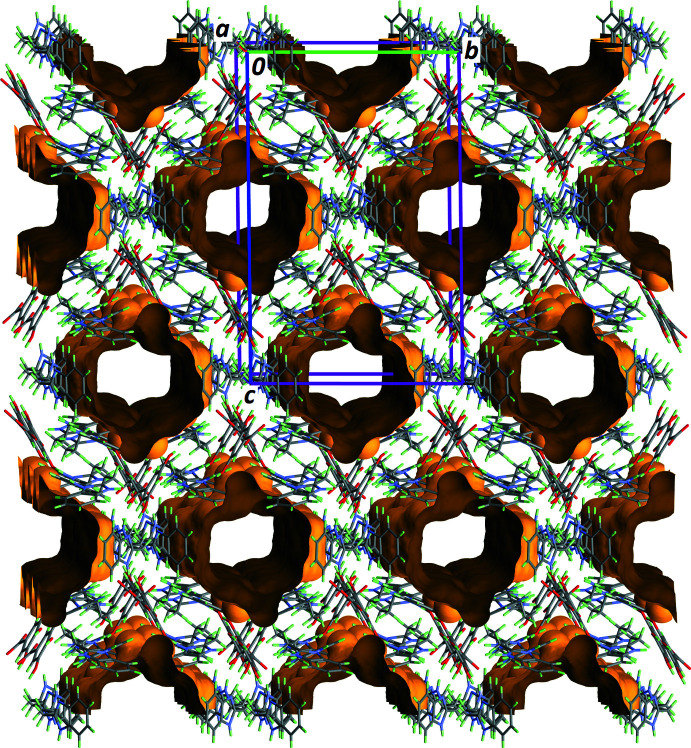
Part of the crystal structure of the title compound showing tunnels in which the disordered ethyl acetate solvent mol­ecules are located (Macrae *et al.*, 2020 ▸). The projection is almost normal to unit-cell axis *a* and the probe radius for the voids is 1.25 Å.

The asymmetric unit of the compound under study contains one cation H*L* ^+^ and one anion Gal^−^ in general positions, and one-half of a mol­ecule *L*, placed on the twofold rotation axis of space group *I*2/*a* (Fig. 1 ▸). The mol­ecular formula is then (H*L* ^+^·Gal^−^)_2_·*L*. With this formula, the calculated Kitaigorodskii packing index (Kitaigorodskii, 1965 ▸), *η* = 0.534, is physically unreasonable, and the refinement can be greatly improved by considering the presence of disordered solvent mol­ecules in the crystal. Large voids of *ca* 2000 Å^3^ per unit cell, which equals 28% of the cell volume, are actually present in the crystal structure, forming wide tunnels running along [100], which are filled with solvent mol­ecules (Fig. 2 ▸). A solvent mask was calculated with *OLEX2* (van der Sluis & Spek, 1990 ▸; Dolomanov *et al.*, 2009 ▸), recovering a density of 564 electrons per unit cell. Since *Z* = 4 for the above-mentioned formula, and considering that only ethyl acetate was used as solvent during the synthesis and crystallization, the formula of the compound was derived as (H*L* ^+^·Gal^−^)_2_·*L*·(C_4_H_8_O_2_)_2.94_. However, it must be noted that the determination of the solvent amount *via* a *SQUEEZE*-like procedure is always inaccurate (*e.g*. Hernández Linares *et al.*, 2016 ▸). The given formula is thus not meant to be precise regarding the overall solvent content. It rather points out that the crystallized compound is a solvated co-crystal between a salt, H*L* ^+^·Gal^−^, and a mol­ecule, *L.*

The presence of voids in the crystal is a consequence of the lack of efficient stacking inter­actions between the co-crystal components, although they contain aromatic heterocycles. This feature is, in turn, related to the different conformations observed for H*L* ^+^ and *L.* The mol­ecule *L* displays a *trans*–*trans* conformation for the propane link bridging the benzimidazole heterocycles: torsion angles C18—C25—C26—C25^i^ and C18^i^—C25^i^—C26^i^—C25 are equal by symmetry, 172.70 (12)° [symmetry code: (i) −*x* + 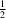 , *y*, −*z* + 1]. In contrast, the cation H*L* ^+^ is placed in a general position, and the propane chain displays a *gauche*–*trans* conformation, reflected in torsion angles C1—C8—C9—C10 = −63.93 (16)° and C11—C10—C9—C8 = 179.45 (11)°. Both *L* and H*L* ^+^ have a bent shape, with dihedral angles between the benzimidazole rings of 65.07 (2) and 37.58 (3)°, respectively. These twisted components do not stack with the gallate anions, probably because, in the first place, the crystal structure is stabilized *via* Coulombic attractions in the ionic part H*L* ^+^·Gal^−^. Only two significant π–π inter­molecular contacts are calculated by *PLATON* (Spek, 2020 ▸), for benzimidazole rings in inversion-related *L* mol­ecules [separation for π-stacked N5/N6/C18/C19/C24 rings: 3.6070 (8) Å, slippage 0.644 Å] and inversion-related H*L* ^+^ cations [separation for π stacking between N1/N2/C1/C2/C7 rings: 3.6672 (7) Å, slippage 0.720 Å]. The gallate anions Gal^−^ are arranged in rows parallel to [100], and do not inter­act with neighbouring aromatic rings: the angles between the Gal^−^ mean plane and surrounding benzimidazole rings are in the range 45.78 (7)–84.96 (6)°. No C—H⋯π inter­actions are observed in the crystal structure.

## 3. Supra­molecular features

**Figure 3:**
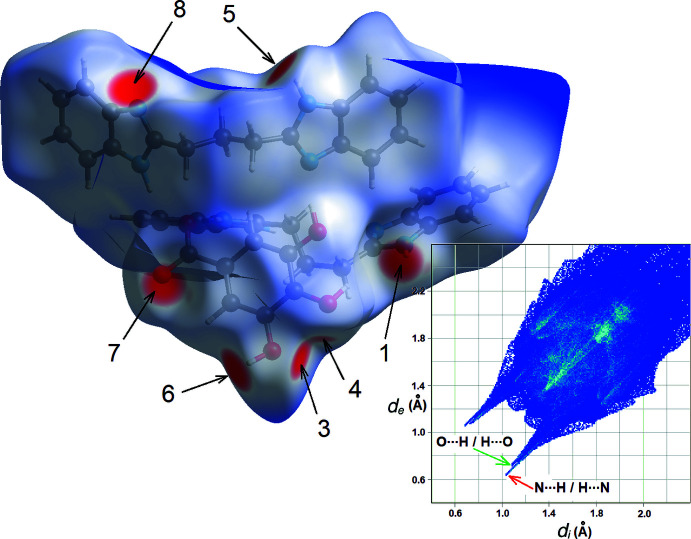
Hirshfeld surface (Spackman *et al.*, 2021 ▸) mapped over *d*_norm_ in the range −0.5 Å (red) to 3.0 Å (blue). Labels 1–8 refer to entries in Table 1 ▸ for each hydrogen bond. Contact N3—H3⋯O1 (entry 2) is not visible, since it corresponds to an intra­molecular hydrogen bond in the inside pocket limited by the Hirshfeld surface. The deep-blue surface at the top of the map is the boundary with the region containing disordered solvent mol­ecules. The two-dimensional fingerprint plot including all contacts is shown in the inset.

Notwithstanding the absence of well-organized stacks in the crystal structure, all N—H, O—H and C=O functional groups are engaged in hydrogen bonds (Table 1 ▸), forming a tri-periodic framework. This is confirmed in the Hirshfeld surface calculated for the expanded asymmetric unit represented in Fig. 1 ▸, that is (H*L* ^+^·Gal^−^)·*L*. This map (Fig. 3 ▸) shows typical spots for regions where inter­atomic distances are shorter than the sum of the van der Waals radii of the atoms. O⋯H and H⋯O contacts account for 16.1% of the Hirshfeld surface, while N⋯H and H⋯N contacts account for 6.0% of the surface. Both kinds of hydrogen bonds generate well-defined spikes in the 2D fingerprint plot, at short (*d*_i_, *d*_e_) coordinates. Apart from these stabilizing inter­actions, the map is dominated by H⋯H contacts (49.3% of the surface) related to van der Waals contacts.

**Figure 4:**
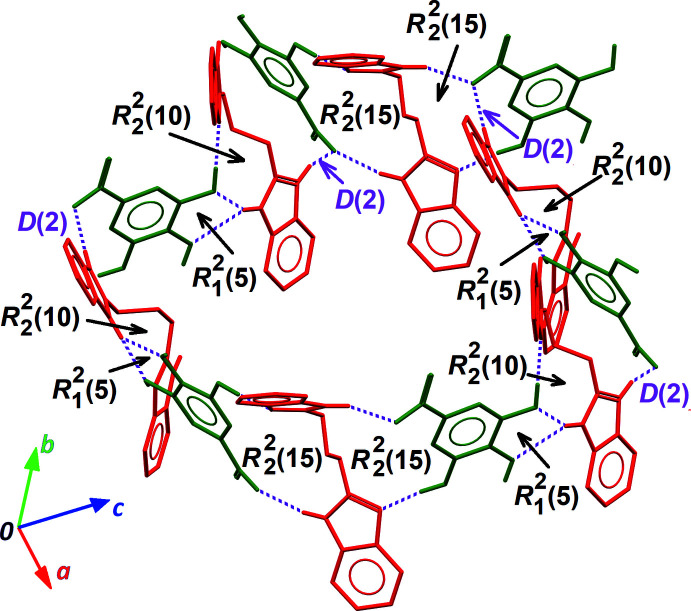
Supra­molecular arrangement of H*L* ^+^, Gal^−^ and *L*, affording the boundary of the cavities containing the disordered solvent. H*L* ^+^ and *L* are coloured red, while Gal^−^ anions are coloured green. Hydrogen bonds are shown as dashed purple lines. All rings (*R*) and discrete (*D*) motifs involved in the building of the supra­molecular ring are indicated. All C-bound H atoms are omitted for clarity.

**Figure 5:**
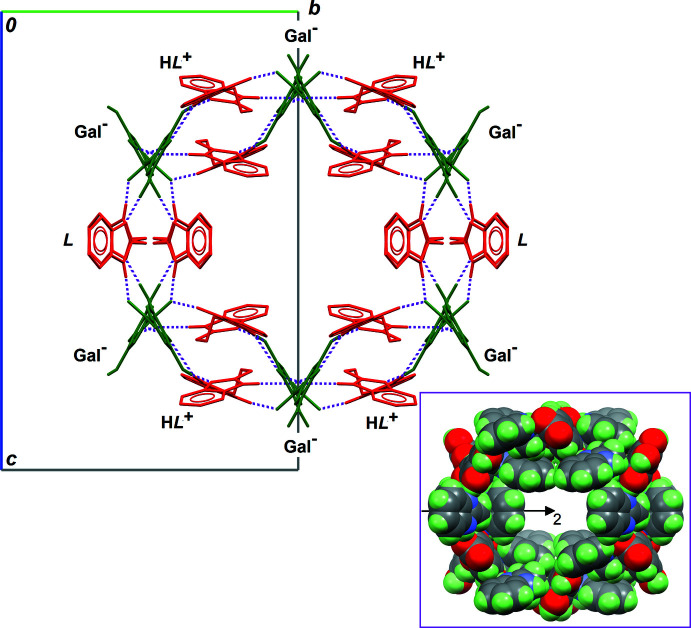
The complete supra­molecular framework enclosing the disordered ethyl acetate solvent, as viewed down the symmetry axis, parallel to [100] in the crystal. The colour code is as for Fig. 4 ▸. All C-bound H atoms are omitted for clarity. The inset is the same framework in a spacefill representation, and including H atoms, showing the real void space available for disordered ethyl acetate mol­ecules. The crystallographic twofold rotation axis position is also shown.

Among the many motifs present in this supra­molecular framework, four are of particular importance for the building of the crystal structure, as they provide the cavities that are filled with disordered solvent mol­ecules. Ring motifs 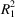 (5), 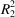 (10) and 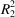 (15) along with discrete motifs *D*(2) link four H*L* ^+^ cations, six Gal^−^ anions and two *L* mol­ecules, forming a ring-shaped supra­molecule (Fig. 4 ▸). Connecting these supra­molecular rings along [100], the remaining hydrogen bonds (entries 3, 4 and 6 in Table 1 ▸, *i.e*. those including ‘−*x* + 1’ in their symmetry operator for the acceptor site) generate the tunnels depicted in Fig. 2 ▸. The boundary of the cavity is formed by a sequence of twelve elements, alternating anions, cations and mol­ecules (Fig. 5 ▸).

The shape of this infinite supra­molecule is close to cylindrical, and its point group is approximately *C*_2*v*_, which is compatible with the space group, *I*2/*a*. However, the crystallographic twofold rotation axis of *I*2/*a* is parallel to [010], and thus it does not coincide with the symmetry axis of the cylindrical supra­molecule, which is parallel to [100]. The most important feature for the crystallization of the title compound is depicted in Fig. 5 ▸: hydro­phobic benzene rings of H*L* ^+^ and *L* point towards the inside of the cylindrical supra­molecule. This arrangement prevents solvent mol­ecules filling these cavities from forming hydrogen bonds with (H*L* ^+^·Gal^−^)_2_·*L*, and, presumably, only weak C—H⋯O=C contacts are present. This explains why ethyl acetate is disordered in this solvated co-crystal.

## 4. Database survey

A search of the CSD (v. 5.43 with all updates; Groom *et al.*, 2016 ▸) shows that crystal-structure determinations of compounds including cations H_2_ *L* ^2+^ or H*L* ^+^ are rather rare. Three salts of H_2_ *L* ^2+^ have been reported so far: H_2_ *L*(SO_4_)·3H_2_O (Clifford *et al.*, 2012 ▸), H_2_ *L*·2(Cl)·2H_2_O (Hu *et al.*, 2006 ▸) and H_2_ *L*(CoCl_4_) (Matthews *et al.*, 2003 ▸). For H*L* ^+^, three crystal structures have also been reported: H*L*(ClO_4_) (Sun *et al.*, 2004 ▸), one co-crystal with trimesic acid and the corresponding carboxyl­ate anion (Feng & Jiang, 2010 ▸), and one Co^II^ complex (Wen *et al.*, 2014 ▸). However, more structures based on the neutral bis-benzimidazole *L* have been deposited in the CSD, with 22 hits, but all are coordination compounds. In particular, it is surprising that the crystal structure of *L* has never been reported.

Regarding the conformation of the cation H*L* ^+^ or the neutral mol­ecule *L*, all possibilities are represented, with central propane bridges found in *trans*–*trans*, *trans*–*gauche* and *gauche*–*gauche* conformations, although the *trans*–*gauche* conformation, observed for H*L* ^+^ in the present complex, is less common, being observed for only one example (Wang & An, 2016 ▸). With such flexibility, almost any relative position for the benzimidazole rings is possible. For the 28 hits retrieved from the CSD, the dihedral angles between benzimidazole rings span a range from 4 to 87°, and the distances between the centroids of the imidazole rings span the range from 3.33 to 5.29 Å.

## 5. Synthesis and crystallization

A solution of 1,3-bis­(1*H*-benzo[*d*]imidazol-2-yl)propane (*L*, 12.4 mg, 0.045 mmol) and gallic acid (HGal, 7.6 mg, 0.045 mmol) in 10 mL of ethyl acetate was heated at boiling temperature until dissolution of the reactants. After filtration, the solution was left at room temperature for slow evaporation of the solvent, giving purple crystals suitable for single-crystal X-ray diffraction analysis.

## 6. Refinement

Crystal data, data collection and structure refinement details are summarized in Table 2 ▸ where the solvent molecules are not considered in the given chemical formula and other crystal data. All H atoms bonded to heteroatoms were refined with free coordinates, in order to achieve an accurate hydrogen-bonding model. Other H atoms were placed in calculated positions. Atom C26 is placed on the twofold rotation axis in space group *I*2/*a*, and therefore, H atoms for this methyl­ene group were modelled with two H atoms (H26*A* and H26*B*) with occupancies of 1/2, in such a way that H26*B* is the image of H26*A* through the symmetry axis and *vice versa* (command HFIX 23 in *SHELXL*; Sheldrick, 2015*b* ▸).

## Supplementary Material

Crystal structure: contains datablock(s) I, global. DOI: 10.1107/S2056989023004279/wm5683sup1.cif

Structure factors: contains datablock(s) I. DOI: 10.1107/S2056989023004279/wm5683Isup2.hkl

CCDC reference: 2263401

Additional supporting information:  crystallographic information; 3D view; checkCIF report

## Figures and Tables

**Table 1:** Hydrogen-bond geometry (Å, °)

*D*—H⋯*A*	*D*—H	H⋯*A*	*D*⋯*A*	*D*—H⋯*A*
N2—H2⋯O2^i^	0.936 (16)	1.924 (17)	2.8204 (14)	159.7 (13)
N3—H3⋯O1	0.880 (16)	1.832 (16)	2.6433 (13)	152.4 (14)
N4—H4⋯O30^ii^	0.867 (16)	2.043 (16)	2.8509 (12)	154.6 (14)
N4—H4⋯O31^ii^	0.867 (16)	2.394 (16)	3.0199 (14)	129.5 (13)
N6—H6*A*⋯O1^iii^	0.893 (15)	1.955 (16)	2.8018 (12)	157.9 (13)
O30—H30⋯N1^iv^	0.946 (18)	1.785 (18)	2.7238 (12)	171.3 (16)
O31—H31⋯O2^i^	0.894 (18)	1.882 (18)	2.7314 (11)	157.8 (16)
O32—H32⋯N5	0.948 (19)	1.717 (19)	2.6515 (14)	167.9 (16)

Symmetry codes: (i) 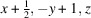 ; (ii) 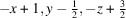 ; (iii) 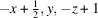 ; (iv) 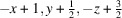 .

**Table 2:** Experimental details

Crystal data	
Chemical formula	2C_17_H_17_N_4_^+^·2C_7_H_5_O_5_^−^·C_17_H_16_N_4_
*M*_r_	1169.25
Crystal system, space group	Monoclinic, *I*2/*a*
Temperature (K)	295
*a*, *b*, *c* (Å)	16.82625 (15), 16.73298 (17), 26.7833 (3)
β (°)	105.2162 (11)
*V* (Å^3^)	7276.57 (14)
*Z*	4
Radiation type	Mo *K*α
μ (mm^−1^)	0.07
Crystal size (mm)	0.60 × 0.48 × 0.37

Data collection	
Diffractometer	Xcalibur, Atlas, Gemini
Absorption correction	Gaussian (*CrysAlis PRO*; Rigaku OD, 2022 ▸)
*T*_min_, *T*_max_	0.761, 1.000
No. of measured, independent and observed [*I* > 2σ(*I*)] reflections	188774, 11088, 8064
*R*_int_	0.064
(sin θ/λ)_max_ (Å^−1^)	0.714

Refinement	
*R*[*F* ^2^ > 2σ(*F* ^2^)], *wR*(*F* ^2^), *S*	0.049, 0.149, 1.06
No. of reflections	11088
No. of parameters	414
H-atom treatment	H atoms treated by a mixture of independent and constrained refinement
Δρ_max_, Δρ_min_ (e Å^−3^)	0.30, −0.18

Computer programs: *CrysAlis PRO* (Rigaku OD, 2022 ▸), *SHELXT2018/2* (Sheldrick, 2015*a* ▸), *SHELXL2018/3* (Sheldrick, 2015*b* ▸), *XP* in *SHELXTL-Plus* (Sheldrick, 2008 ▸), *Mercury* (Macrae *et al.*, 2020 ▸), CrystalExplorer (Spackman *et al.*, 2021 ▸) and *publCIF* (Westrip, 2010 ▸).

## supplementary crystallographic information

### Crystal data

**Table:**

2C_17_H_17_N_4_^+^·2C_7_H_5_O_5_^−^·C_17_H_16_N_4_	*F*(000) = 2456
*M**_r_* = 1169.25	*D*_x_ = 1.067 Mg m^−^^3^
Monoclinic, *I*2/*a*	Mo *K*α radiation, λ = 0.71073 Å
*a* = 16.82625 (15) Å	Cell parameters from 53060 reflections
*b* = 16.73298 (17) Å	θ = 1.5–33.7°
*c* = 26.7833 (3) Å	µ = 0.07 mm^−^^1^
β = 105.2162 (11)°	*T* = 295 K
*V* = 7276.57 (14) Å^3^	Block, purple
*Z* = 4	0.60 × 0.48 × 0.37 mm

### Data collection

**Table:**

Xcalibur, Atlas, Gemini diffractometer	11088 independent reflections
Radiation source: fine-focus sealed X-ray tube, Enhance (Mo) X-ray Source	8064 reflections with *I* > 2σ(*I*)
Graphite monochromator	*R*_int_ = 0.064
Detector resolution: 10.5564 pixels mm^-1^	θ_max_ = 30.5°, θ_min_ = 1.6°
ω scans	*h* = −24→24
Absorption correction: gaussian (CrysAlisPro; Rigaku OD, 2022)	*k* = −23→23
*T*_min_ = 0.761, *T*_max_ = 1.000	*l* = −38→38
188774 measured reflections	

### Refinement

**Table:**

Refinement on *F*^2^	Primary atom site location: dual
Least-squares matrix: full	Secondary atom site location: difference Fourier map
*R*[*F*^2^ > 2σ(*F*^2^)] = 0.049	Hydrogen site location: mixed
*wR*(*F*^2^) = 0.149	H atoms treated by a mixture of independent and constrained refinement
*S* = 1.06	*w* = 1/[σ^2^(*F*_o_^2^) + (0.0782*P*)^2^ + 1.8337*P*] where *P* = (*F*_o_^2^ + 2*F*_c_^2^)/3
11088 reflections	(Δ/σ)_max_ = 0.001
414 parameters	Δρ_max_ = 0.30 e Å^−^^3^
0 restraints	Δρ_min_ = −0.18 e Å^−^^3^
0 constraints	

### Fractional atomic coordinates and isotropic or equivalent isotropic displacement parameters (Å^2^)

**Table:**

	*x*	*y*	*z*	*U*_iso_*/*U*_eq_	Occ. (<1)
N1	0.62719 (6)	0.20714 (6)	0.69126 (4)	0.0409 (2)	
N2	0.70426 (6)	0.31366 (7)	0.68709 (4)	0.0415 (2)	
H2	0.7153 (9)	0.3686 (10)	0.6889 (6)	0.050*	
N3	0.29155 (6)	0.28875 (6)	0.65905 (4)	0.0410 (2)	
H3	0.2821 (9)	0.3375 (10)	0.6465 (6)	0.049*	
N4	0.35419 (6)	0.17996 (6)	0.69126 (5)	0.0456 (3)	
H4	0.3956 (10)	0.1491 (10)	0.7050 (6)	0.055*	
C1	0.63565 (6)	0.28528 (7)	0.69822 (4)	0.0369 (2)	
C2	0.69506 (7)	0.18377 (8)	0.67405 (5)	0.0417 (3)	
C3	0.71780 (10)	0.10890 (10)	0.66007 (7)	0.0588 (4)	
H3A	0.686161	0.063838	0.661591	0.071*	
C4	0.78909 (12)	0.10380 (12)	0.64384 (8)	0.0715 (5)	
H4A	0.805713	0.054293	0.634429	0.086*	
C5	0.83663 (11)	0.17096 (12)	0.64124 (7)	0.0710 (5)	
H5	0.884062	0.165163	0.629939	0.085*	
C6	0.81553 (9)	0.24544 (11)	0.65486 (6)	0.0586 (4)	
H6	0.847519	0.290187	0.653195	0.070*	
C7	0.74379 (7)	0.25066 (8)	0.67130 (5)	0.0420 (3)	
C8	0.58072 (7)	0.34195 (8)	0.71577 (6)	0.0454 (3)	
H8A	0.570693	0.387335	0.692415	0.055*	
H8B	0.609617	0.361623	0.749748	0.055*	
C9	0.49824 (7)	0.30894 (8)	0.71883 (5)	0.0458 (3)	
H9A	0.469890	0.349272	0.733691	0.055*	
H9B	0.507561	0.263106	0.741808	0.055*	
C10	0.44301 (7)	0.28374 (8)	0.66621 (6)	0.0454 (3)	
H10A	0.432614	0.329527	0.643194	0.055*	
H10B	0.471005	0.243466	0.651070	0.055*	
C11	0.36365 (7)	0.25125 (7)	0.67136 (5)	0.0410 (3)	
C12	0.23293 (7)	0.24029 (7)	0.67147 (5)	0.0403 (3)	
C13	0.14945 (8)	0.25199 (9)	0.66602 (6)	0.0542 (4)	
H13	0.123234	0.299434	0.653072	0.065*	
C14	0.10775 (9)	0.18973 (11)	0.68079 (7)	0.0650 (4)	
H14	0.051424	0.194600	0.677015	0.078*	
C15	0.14755 (9)	0.11917 (10)	0.70137 (7)	0.0637 (4)	
H15	0.116961	0.078516	0.711040	0.076*	
C16	0.23059 (8)	0.10804 (9)	0.70774 (6)	0.0536 (3)	
H16	0.257062	0.061314	0.721899	0.064*	
C17	0.27263 (7)	0.17010 (8)	0.69195 (5)	0.0422 (3)	
N5	0.45349 (6)	0.41770 (7)	0.53870 (4)	0.0448 (3)	
N6	0.41648 (6)	0.41115 (7)	0.45294 (4)	0.0386 (2)	
H6A	0.3833 (9)	0.4196 (9)	0.4214 (6)	0.046*	
C18	0.39799 (6)	0.43829 (7)	0.49600 (4)	0.0363 (2)	
C19	0.51184 (7)	0.37344 (8)	0.52212 (5)	0.0427 (3)	
C20	0.58383 (9)	0.33716 (11)	0.55080 (6)	0.0620 (4)	
H20	0.599317	0.339769	0.586743	0.074*	
C21	0.63136 (10)	0.29722 (12)	0.52407 (7)	0.0707 (5)	
H21	0.679105	0.271436	0.542360	0.085*	
C22	0.60934 (9)	0.29471 (11)	0.47023 (7)	0.0655 (4)	
H22	0.643155	0.267777	0.453395	0.079*	
C23	0.53885 (8)	0.33102 (10)	0.44121 (6)	0.0533 (3)	
H23	0.524628	0.329751	0.405239	0.064*	
C24	0.48992 (6)	0.36969 (8)	0.46827 (5)	0.0392 (3)	
C25	0.32225 (6)	0.48543 (8)	0.49463 (5)	0.0398 (3)	
H25A	0.306067	0.514498	0.462208	0.048*	
H25B	0.334742	0.524315	0.522463	0.048*	
C26	0.250000	0.43372 (11)	0.500000	0.0390 (3)	
H26A	0.232262	0.399658	0.469816	0.047*	0.5
H26B	0.267739	0.399662	0.530185	0.047*	0.5
C27	0.24259 (6)	0.48453 (6)	0.66505 (4)	0.0286 (2)	
O1	0.22012 (4)	0.42873 (5)	0.63255 (3)	0.03592 (18)	
O2	0.19661 (4)	0.51802 (5)	0.68876 (4)	0.0406 (2)	
C28	0.33088 (5)	0.51107 (6)	0.67702 (4)	0.0279 (2)	
C29	0.36507 (6)	0.55315 (6)	0.72219 (4)	0.0301 (2)	
H29	0.332142	0.568311	0.743553	0.036*	
C30	0.44827 (6)	0.57278 (6)	0.73570 (4)	0.0304 (2)	
O30	0.48495 (5)	0.61169 (5)	0.78071 (4)	0.0434 (2)	
H30	0.4435 (11)	0.6401 (11)	0.7912 (6)	0.065*	
C31	0.49785 (6)	0.55062 (6)	0.70354 (4)	0.0305 (2)	
O31	0.57953 (4)	0.56927 (6)	0.71834 (4)	0.0422 (2)	
H31	0.6076 (11)	0.5401 (10)	0.7008 (7)	0.063*	
C32	0.46247 (6)	0.51162 (7)	0.65697 (4)	0.0307 (2)	
O32	0.51369 (5)	0.49489 (6)	0.62700 (3)	0.0442 (2)	
H32	0.4854 (11)	0.4668 (11)	0.5968 (7)	0.066*	
C33	0.37932 (6)	0.49074 (7)	0.64409 (4)	0.0307 (2)	
H33	0.356292	0.463270	0.613533	0.037*	

### Atomic displacement parameters (Å^2^)

**Table:**

	*U*^11^	*U*^22^	*U*^33^	*U*^12^	*U*^13^	*U*^23^
N1	0.0334 (5)	0.0412 (5)	0.0503 (6)	−0.0029 (4)	0.0148 (4)	0.0086 (4)
N2	0.0328 (5)	0.0429 (5)	0.0490 (6)	−0.0068 (4)	0.0112 (4)	0.0071 (4)
N3	0.0297 (4)	0.0356 (5)	0.0566 (6)	0.0051 (4)	0.0091 (4)	0.0087 (4)
N4	0.0278 (4)	0.0397 (5)	0.0654 (7)	0.0072 (4)	0.0054 (4)	0.0122 (5)
C1	0.0278 (5)	0.0421 (6)	0.0392 (6)	−0.0040 (4)	0.0059 (4)	0.0085 (5)
C2	0.0363 (6)	0.0467 (6)	0.0437 (6)	−0.0009 (5)	0.0132 (5)	0.0077 (5)
C3	0.0602 (9)	0.0508 (8)	0.0708 (10)	0.0004 (7)	0.0269 (8)	0.0013 (7)
C4	0.0716 (11)	0.0710 (11)	0.0812 (12)	0.0131 (9)	0.0368 (9)	−0.0051 (9)
C5	0.0561 (9)	0.0932 (13)	0.0751 (11)	0.0046 (9)	0.0373 (8)	−0.0008 (10)
C6	0.0445 (7)	0.0755 (10)	0.0628 (9)	−0.0070 (7)	0.0264 (7)	0.0043 (8)
C7	0.0346 (5)	0.0511 (7)	0.0410 (6)	−0.0033 (5)	0.0112 (5)	0.0090 (5)
C8	0.0349 (6)	0.0421 (6)	0.0598 (8)	−0.0039 (5)	0.0132 (5)	−0.0001 (6)
C9	0.0343 (6)	0.0468 (7)	0.0581 (8)	−0.0011 (5)	0.0155 (5)	0.0017 (6)
C10	0.0303 (5)	0.0464 (7)	0.0587 (8)	0.0019 (5)	0.0101 (5)	0.0086 (6)
C11	0.0274 (5)	0.0401 (6)	0.0524 (7)	0.0047 (4)	0.0051 (5)	0.0052 (5)
C12	0.0293 (5)	0.0403 (6)	0.0501 (7)	0.0040 (4)	0.0084 (5)	0.0056 (5)
C13	0.0334 (6)	0.0585 (8)	0.0719 (9)	0.0114 (6)	0.0159 (6)	0.0165 (7)
C14	0.0340 (6)	0.0784 (11)	0.0854 (11)	0.0052 (6)	0.0205 (7)	0.0242 (9)
C15	0.0445 (7)	0.0668 (10)	0.0807 (11)	−0.0059 (7)	0.0181 (7)	0.0238 (8)
C16	0.0434 (7)	0.0478 (7)	0.0665 (9)	0.0008 (6)	0.0093 (6)	0.0170 (6)
C17	0.0304 (5)	0.0422 (6)	0.0505 (7)	0.0031 (4)	0.0047 (5)	0.0062 (5)
N5	0.0292 (4)	0.0697 (7)	0.0353 (5)	0.0067 (4)	0.0078 (4)	−0.0058 (5)
N6	0.0260 (4)	0.0542 (6)	0.0341 (5)	0.0023 (4)	0.0056 (4)	−0.0030 (4)
C18	0.0233 (4)	0.0482 (6)	0.0383 (6)	−0.0028 (4)	0.0096 (4)	−0.0035 (5)
C19	0.0271 (5)	0.0609 (8)	0.0392 (6)	0.0048 (5)	0.0074 (4)	−0.0040 (5)
C20	0.0398 (7)	0.0968 (12)	0.0447 (7)	0.0201 (7)	0.0025 (6)	0.0013 (8)
C21	0.0443 (7)	0.0944 (13)	0.0682 (10)	0.0301 (8)	0.0057 (7)	−0.0013 (9)
C22	0.0461 (7)	0.0837 (11)	0.0679 (10)	0.0210 (7)	0.0172 (7)	−0.0135 (8)
C23	0.0434 (7)	0.0711 (9)	0.0458 (7)	0.0088 (6)	0.0126 (5)	−0.0130 (6)
C24	0.0274 (5)	0.0507 (7)	0.0384 (6)	0.0014 (4)	0.0066 (4)	−0.0054 (5)
C25	0.0251 (5)	0.0489 (7)	0.0473 (7)	0.0008 (4)	0.0130 (4)	−0.0009 (5)
C26	0.0233 (6)	0.0469 (9)	0.0475 (9)	0.000	0.0106 (6)	0.000
C27	0.0185 (4)	0.0291 (5)	0.0372 (5)	0.0008 (3)	0.0053 (4)	0.0035 (4)
O1	0.0221 (3)	0.0364 (4)	0.0450 (4)	−0.0009 (3)	0.0012 (3)	−0.0033 (3)
O2	0.0209 (3)	0.0428 (4)	0.0608 (5)	−0.0012 (3)	0.0156 (3)	−0.0077 (4)
C28	0.0174 (4)	0.0298 (5)	0.0366 (5)	0.0004 (3)	0.0070 (3)	0.0014 (4)
C29	0.0203 (4)	0.0318 (5)	0.0403 (5)	0.0004 (3)	0.0116 (4)	−0.0040 (4)
C30	0.0217 (4)	0.0303 (5)	0.0391 (5)	−0.0007 (3)	0.0078 (4)	−0.0061 (4)
O30	0.0251 (4)	0.0513 (5)	0.0539 (5)	−0.0036 (3)	0.0104 (3)	−0.0250 (4)
C31	0.0180 (4)	0.0323 (5)	0.0418 (6)	−0.0009 (3)	0.0086 (4)	−0.0024 (4)
O31	0.0183 (3)	0.0525 (5)	0.0571 (5)	−0.0054 (3)	0.0124 (3)	−0.0179 (4)
C32	0.0202 (4)	0.0394 (5)	0.0340 (5)	0.0008 (4)	0.0096 (4)	−0.0001 (4)
O32	0.0231 (3)	0.0740 (6)	0.0385 (4)	−0.0028 (4)	0.0137 (3)	−0.0109 (4)
C33	0.0210 (4)	0.0388 (5)	0.0317 (5)	−0.0007 (4)	0.0057 (4)	−0.0024 (4)

### Geometric parameters (Å, º)

**Table:**

N1—C1	1.3230 (16)	C16—H16	0.9300
N1—C2	1.3950 (15)	N5—C18	1.3184 (15)
N2—C1	1.3520 (14)	N5—C19	1.3922 (15)
N2—C7	1.3712 (17)	N6—C18	1.3496 (15)
N2—H2	0.936 (16)	N6—C24	1.3822 (14)
N3—C11	1.3282 (14)	N6—H6A	0.893 (15)
N3—C12	1.3831 (15)	C18—C25	1.4910 (15)
N3—H3	0.880 (16)	C19—C20	1.3920 (18)
N4—C11	1.3330 (16)	C19—C24	1.3933 (17)
N4—C17	1.3872 (15)	C20—C21	1.378 (2)
N4—H4	0.867 (16)	C20—H20	0.9300
C1—C8	1.4841 (18)	C21—C22	1.392 (2)
C2—C3	1.390 (2)	C21—H21	0.9300
C2—C7	1.4007 (17)	C22—C23	1.377 (2)
C3—C4	1.382 (2)	C22—H22	0.9300
C3—H3A	0.9300	C23—C24	1.3909 (17)
C4—C5	1.392 (3)	C23—H23	0.9300
C4—H4A	0.9300	C25—C26	1.5293 (15)
C5—C6	1.371 (3)	C25—H25A	0.9700
C5—H5	0.9300	C25—H25B	0.9700
C6—C7	1.3921 (18)	C26—H26A	0.9700
C6—H6	0.9300	C26—H26B	0.9700
C8—C9	1.5158 (16)	C27—O2	1.2545 (12)
C8—H8A	0.9700	C27—O1	1.2648 (13)
C8—H8B	0.9700	C27—C28	1.5024 (13)
C9—C10	1.5298 (19)	C28—C29	1.3876 (14)
C9—H9A	0.9700	C28—C33	1.3910 (14)
C9—H9B	0.9700	C29—C30	1.3903 (13)
C10—C11	1.4814 (16)	C29—H29	0.9300
C10—H10A	0.9700	C30—O30	1.3665 (13)
C10—H10B	0.9700	C30—C31	1.3973 (14)
C12—C13	1.3874 (16)	O30—H30	0.946 (18)
C12—C17	1.3909 (16)	C31—O31	1.3629 (11)
C13—C14	1.371 (2)	C31—C32	1.3953 (15)
C13—H13	0.9300	O31—H31	0.894 (18)
C14—C15	1.397 (2)	C32—O32	1.3521 (12)
C14—H14	0.9300	C32—C33	1.3946 (13)
C15—C16	1.375 (2)	O32—H32	0.948 (19)
C15—H15	0.9300	C33—H33	0.9300
C16—C17	1.3838 (18)		
			
C1—N1—C2	104.86 (10)	C17—C16—H16	121.8
C1—N2—C7	108.02 (10)	C16—C17—N4	132.87 (11)
C1—N2—H2	120.2 (9)	C16—C17—C12	121.54 (11)
C7—N2—H2	131.7 (9)	N4—C17—C12	105.59 (10)
C11—N3—C12	109.02 (10)	C18—N5—C19	105.11 (10)
C11—N3—H3	126.4 (10)	C18—N6—C24	107.69 (10)
C12—N3—H3	124.5 (10)	C18—N6—H6A	121.8 (9)
C11—N4—C17	109.51 (10)	C24—N6—H6A	130.4 (9)
C11—N4—H4	122.3 (11)	N5—C18—N6	112.55 (10)
C17—N4—H4	127.7 (11)	N5—C18—C25	124.47 (10)
N1—C1—N2	112.50 (11)	N6—C18—C25	122.98 (10)
N1—C1—C8	128.54 (10)	C20—C19—N5	129.82 (12)
N2—C1—C8	118.96 (11)	C20—C19—C24	120.47 (12)
C3—C2—N1	130.46 (12)	N5—C19—C24	109.69 (10)
C3—C2—C7	119.99 (12)	C21—C20—C19	117.68 (13)
N1—C2—C7	109.55 (11)	C21—C20—H20	121.2
C4—C3—C2	117.62 (15)	C19—C20—H20	121.2
C4—C3—H3A	121.2	C20—C21—C22	121.31 (14)
C2—C3—H3A	121.2	C20—C21—H21	119.3
C3—C4—C5	121.58 (16)	C22—C21—H21	119.3
C3—C4—H4A	119.2	C23—C22—C21	121.81 (14)
C5—C4—H4A	119.2	C23—C22—H22	119.1
C6—C5—C4	121.89 (14)	C21—C22—H22	119.1
C6—C5—H5	119.1	C22—C23—C24	116.82 (13)
C4—C5—H5	119.1	C22—C23—H23	121.6
C5—C6—C7	116.60 (15)	C24—C23—H23	121.6
C5—C6—H6	121.7	N6—C24—C23	133.16 (11)
C7—C6—H6	121.7	N6—C24—C19	104.94 (10)
N2—C7—C6	132.60 (13)	C23—C24—C19	121.89 (11)
N2—C7—C2	105.08 (10)	C18—C25—C26	113.19 (11)
C6—C7—C2	122.32 (13)	C18—C25—H25A	108.9
C1—C8—C9	116.07 (11)	C26—C25—H25A	108.9
C1—C8—H8A	108.3	C18—C25—H25B	108.9
C9—C8—H8A	108.3	C26—C25—H25B	108.9
C1—C8—H8B	108.3	H25A—C25—H25B	107.8
C9—C8—H8B	108.3	C25^i^—C26—C25	111.10 (14)
H8A—C8—H8B	107.4	C25^i^—C26—H26A	109.4
C8—C9—C10	113.20 (11)	C25—C26—H26A	109.4
C8—C9—H9A	108.9	C25^i^—C26—H26B	109.4
C10—C9—H9A	108.9	C25—C26—H26B	109.4
C8—C9—H9B	108.9	H26A—C26—H26B	108.0
C10—C9—H9B	108.9	O2—C27—O1	124.43 (9)
H9A—C9—H9B	107.8	O2—C27—C28	117.82 (9)
C11—C10—C9	111.09 (11)	O1—C27—C28	117.73 (9)
C11—C10—H10A	109.4	C29—C28—C33	119.94 (9)
C9—C10—H10A	109.4	C29—C28—C27	119.76 (9)
C11—C10—H10B	109.4	C33—C28—C27	120.24 (9)
C9—C10—H10B	109.4	C28—C29—C30	120.36 (9)
H10A—C10—H10B	108.0	C28—C29—H29	119.8
N3—C11—N4	108.96 (10)	C30—C29—H29	119.8
N3—C11—C10	126.19 (11)	O30—C30—C29	122.21 (9)
N4—C11—C10	124.80 (10)	O30—C30—C31	117.75 (8)
N3—C12—C13	131.16 (12)	C29—C30—C31	120.02 (9)
N3—C12—C17	106.91 (10)	C30—O30—H30	107.7 (11)
C13—C12—C17	121.92 (12)	O31—C31—C32	121.88 (9)
C14—C13—C12	116.31 (13)	O31—C31—C30	118.67 (9)
C14—C13—H13	121.8	C32—C31—C30	119.44 (9)
C12—C13—H13	121.8	C31—O31—H31	110.8 (11)
C13—C14—C15	121.82 (13)	O32—C32—C33	123.76 (10)
C13—C14—H14	119.1	O32—C32—C31	116.02 (9)
C15—C14—H14	119.1	C33—C32—C31	120.21 (9)
C16—C15—C14	121.98 (14)	C32—O32—H32	110.7 (11)
C16—C15—H15	119.0	C28—C33—C32	119.91 (10)
C14—C15—H15	119.0	C28—C33—H33	120.0
C15—C16—C17	116.41 (13)	C32—C33—H33	120.0
C15—C16—H16	121.8		
			
C2—N1—C1—N2	−0.44 (14)	C13—C12—C17—N4	179.89 (13)
C2—N1—C1—C8	179.31 (12)	C19—N5—C18—N6	−0.52 (15)
C7—N2—C1—N1	0.51 (14)	C19—N5—C18—C25	178.77 (11)
C7—N2—C1—C8	−179.26 (11)	C24—N6—C18—N5	−0.21 (14)
C1—N1—C2—C3	−179.18 (15)	C24—N6—C18—C25	−179.51 (11)
C1—N1—C2—C7	0.21 (14)	C18—N5—C19—C20	179.82 (16)
N1—C2—C3—C4	179.44 (15)	C18—N5—C19—C24	1.06 (15)
C7—C2—C3—C4	0.1 (2)	N5—C19—C20—C21	−179.31 (16)
C2—C3—C4—C5	−0.3 (3)	C24—C19—C20—C21	−0.7 (2)
C3—C4—C5—C6	0.3 (3)	C19—C20—C21—C22	1.5 (3)
C4—C5—C6—C7	−0.2 (3)	C20—C21—C22—C23	−0.8 (3)
C1—N2—C7—C6	179.15 (14)	C21—C22—C23—C24	−0.7 (3)
C1—N2—C7—C2	−0.35 (13)	C18—N6—C24—C23	−178.24 (15)
C5—C6—C7—N2	−179.35 (15)	C18—N6—C24—C19	0.84 (14)
C5—C6—C7—C2	0.1 (2)	C22—C23—C24—N6	−179.50 (15)
C3—C2—C7—N2	179.55 (13)	C22—C23—C24—C19	1.5 (2)
N1—C2—C7—N2	0.09 (14)	C20—C19—C24—N6	179.93 (14)
C3—C2—C7—C6	0.0 (2)	N5—C19—C24—N6	−1.18 (15)
N1—C2—C7—C6	−179.47 (12)	C20—C19—C24—C23	−0.9 (2)
N1—C1—C8—C9	−9.7 (2)	N5—C19—C24—C23	178.03 (13)
N2—C1—C8—C9	170.03 (11)	N5—C18—C25—C26	−86.02 (14)
C1—C8—C9—C10	−63.93 (16)	N6—C18—C25—C26	93.19 (13)
C8—C9—C10—C11	179.45 (11)	C18—C25—C26—C25^i^	172.70 (12)
C12—N3—C11—N4	0.14 (15)	O2—C27—C28—C29	18.00 (15)
C12—N3—C11—C10	−177.42 (13)	O1—C27—C28—C29	−160.52 (10)
C17—N4—C11—N3	0.16 (16)	O2—C27—C28—C33	−164.68 (10)
C17—N4—C11—C10	177.76 (13)	O1—C27—C28—C33	16.80 (15)
C9—C10—C11—N3	103.86 (15)	C33—C28—C29—C30	−2.24 (16)
C9—C10—C11—N4	−73.33 (17)	C27—C28—C29—C30	175.09 (9)
C11—N3—C12—C13	−179.73 (15)	C28—C29—C30—O30	−177.66 (10)
C11—N3—C12—C17	−0.38 (15)	C28—C29—C30—C31	0.45 (16)
N3—C12—C13—C14	177.75 (15)	O30—C30—C31—O31	−0.48 (16)
C17—C12—C13—C14	−1.5 (2)	C29—C30—C31—O31	−178.67 (10)
C12—C13—C14—C15	1.5 (3)	O30—C30—C31—C32	−179.25 (10)
C13—C14—C15—C16	−0.3 (3)	C29—C30—C31—C32	2.55 (16)
C14—C15—C16—C17	−0.9 (3)	O31—C31—C32—O32	−1.24 (16)
C15—C16—C17—N4	−178.51 (16)	C30—C31—C32—O32	177.49 (10)
C15—C16—C17—C12	0.9 (2)	O31—C31—C32—C33	177.48 (10)
C11—N4—C17—C16	179.11 (16)	C30—C31—C32—C33	−3.78 (16)
C11—N4—C17—C12	−0.39 (15)	C29—C28—C33—C32	1.01 (16)
N3—C12—C17—C16	−179.11 (13)	C27—C28—C33—C32	−176.32 (9)
C13—C12—C17—C16	0.3 (2)	O32—C32—C33—C28	−179.36 (10)
N3—C12—C17—N4	0.46 (15)	C31—C32—C33—C28	2.02 (16)

Symmetry code: (i) −*x*+1/2, *y*, −*z*+1.

### Hydrogen-bond geometry (Å, º)

**Table:**

*D*—H···*A*	*D*—H	H···*A*	*D*···*A*	*D*—H···*A*
N2—H2···O2^ii^	0.936 (16)	1.924 (17)	2.8204 (14)	159.7 (13)
N3—H3···O1	0.880 (16)	1.832 (16)	2.6433 (13)	152.4 (14)
N4—H4···O30^iii^	0.867 (16)	2.043 (16)	2.8509 (12)	154.6 (14)
N4—H4···O31^iii^	0.867 (16)	2.394 (16)	3.0199 (14)	129.5 (13)
N6—H6*A*···O1^i^	0.893 (15)	1.955 (16)	2.8018 (12)	157.9 (13)
O30—H30···N1^iv^	0.946 (18)	1.785 (18)	2.7238 (12)	171.3 (16)
O31—H31···O2^ii^	0.894 (18)	1.882 (18)	2.7314 (11)	157.8 (16)
O32—H32···N5	0.948 (19)	1.717 (19)	2.6515 (14)	167.9 (16)
C8—H8*A*···O32	0.97	2.52	3.4736 (17)	168
C10—H10*B*···N1	0.97	2.64	3.2566 (16)	122

Symmetry codes: (i) −*x*+1/2, *y*, −*z*+1; (ii) *x*+1/2, −*y*+1, *z*; (iii) −*x*+1, *y*−1/2, −*z*+3/2; (iv) −*x*+1, *y*+1/2, −*z*+3/2.
